# Guided Bone Regeneration with Ammoniomethacrylate-Based Barrier Membranes in a Radial Defect Model

**DOI:** 10.1155/2020/5905740

**Published:** 2020-10-13

**Authors:** David Kirmayer, Ada Grin, Julia Gefter (Shenderovich), Michael Friedman, Jacob Rachmilewitz, Rami Mosheiff, Ron Kenett, Amal Khoury

**Affiliations:** ^1^Department of Pharmaceutics, Institute for Drug Research, School of Pharmacy, Faculty of Medicine, The Hebrew University of Jerusalem, 91120, Israel; ^2^Goldyne Savad Institute of Gene Therapy, Hadassah-Hebrew University Medical Center, Jerusalem 91120, Israel; ^3^Department of Orthopedic Surgery, Hadassah-Hebrew University Medical Center, Jerusalem 91120, Israel; ^4^Giuseppe Peano Department of Mathematics, University of Turin, 10123 Torino, Italy

## Abstract

Large bone defects pose an unsolved challenge for orthopedic surgeons. Our group has previously reported the construction of a barrier membrane made of ammoniomethacrylate copolymer USP (AMCA), which supports the adhesion, proliferation, and osteoblastic differentiation of human mesenchymal stem cells (hMSCs). In this study, we report the use of AMCA membranes to seclude critical segmental defect (~1.0 cm) created in the middle third of rabbit radius and test the efficiency of bone regeneration. Bone regeneration was assessed by radiography, biweekly for 8 weeks. The results were verified by histology and micro-CT at the end of the follow-up. The AMCA membranes were found superior to no treatment in terms of new bone formation in the defect, bone volume, callus surface area normalized to total volume, and the number of bone trabeculae, after eight weeks. Additional factors were then assessed, and these included the addition of simvastatin to the membrane, coating the membrane with human MSC, and a combination of those. The addition of simvastatin to the membranes demonstrated a stronger effect at a similar radiological follow-up. We conclude that AMCA barrier membranes *per se* and simvastatin delivered in a controlled manner improve bone regeneration outcome.

## 1. Introduction

Despite the great regenerative potential of the bone, it may sometimes be insufficient and large segmental bone defects are frequently encountered in the treatment of high-energy fractures, osteomyelitis, and revision arthroplasty and following tumor resection. Critical defects may be predictive of nonunion, and a rule of thumb is that a defect that is larger than 1.5-2 times the diaphysis diameter may be considered critical [1]. Generally speaking, regeneration of bone defects occurs naturally during fracture healing; and yet, clinical management strategies available today for large segmental bone defects suffer from several drawbacks. These strategies include autografts, allografts, distraction osteogenesis, and various osteogenic and osteoconductive substances, including many experimental treatments. Many materials employed in these approaches have been recently reviewed, including general reviews [2], scaffold approaches [3], composite materials [4], hydrogels [5], grafts [6], mesenchymal stem cell (MSC) transplantation [7–10], and barrier membrane approach [1, 11, 12]. Mesenchymal stem cells constitute a heterogeneous multipotent stem cell population with similar characteristics and are distributed in multiple tissues, including periosteum, and they can differentiate into chondroblasts, lipoblasts, and osteoblasts and moreover may also generate mature cells typically arising from the endoderm and ectoderm. The role of improved periosteum function was also regarded as a model for guided bone regeneration [13].

The biological rationale behind barrier membranes is the mechanical exclusion of undesirable soft tissues from ingrowing into the osseous defect, thereby allowing only osteogenic cell populations derived from the parent bone to repopulate the osseous wound space [14, 15]. The desirable characteristics of barrier membranes include biocompatibility, defect occlusion properties, integration by host tissues, clinical manageability, and space-making ability [1, 16]. Different barrier membranes have been developed and investigated to combat nonunion fractures, but none of these arrived into routine clinical practice. The main types of membranes and their drawbacks have been discussed elsewhere, including expanded polytetrafluoroethylene (e-PTFE) [17–19], aliphatic polyesters [20–29], and collagen [30–33].

Our group has previously reported a membrane based on ammoniomethacrylate copolymer, type A, USP (AMCA). The membrane supports the adhesion, proliferation, and osteoblastic differentiation of human MSCs [34] and is suitable for controlled release of various drugs, including simvastatin [35]. Simvastatin (PubChem CID: 54454) and other statins were shown to enhance new bone formation via bone morphogenic protein-2-like (BMP-2) activity [36]. The membrane was characterized additionally as a thin, flexible, nanoporous (ca. 180 nm) polymeric sheet, and for all these reasons, it is deemed suitable for use as a barrier membrane.

In this work, we tested the efficiency of AMCA membranes; neat membranes, simvastatin-eluting membranes and hMSC-coated membranes were evaluated for the treatment of large bone defects defect, using the critical radial defect model in rabbits [24, 37–40], which is an acceptable model for the study of nonunion. The defect healing was followed by radiology for 8 weeks, at time points 2, 4, 6, and 8 weeks. After 8 weeks, micro-CT and histology were performed.

## 2. Materials and Methods

### 2.1. Experiment Design

All experiments were approved by the institutional ethics board (Hadassah Medical Center) at the time they were performed. Mature male New Zealand rabbits (obtained from Harlan Laboratories, Rehovot, Israel, weighing 2.5-4.0 kg) were used. The animals were assigned to five different treatment groups over time.

First, in a group of 6 animals, the efficacy of neat AMCA membrane alone versus no treatment in contralateral limb defect was evaluated (designated “AMCA/0” herein); i.e., both of the forelimbs were operated, one was left randomly without treatment, and the contralateral was treated with the membrane. The minimal sample size was chosen and maintained from ethical considerations and was corroborated by the power calculation, as described below.

In another group of 10 animals (6 + 4), the evaluated effect was coating of the membrane with hMSC versus membrane alone (designated “hMSC/AMCA”), in similar crossover approach.

As the ethical practice changed at that time to the modern approach perceiving the two operated limbs as excessive suffering, further experiments were conducted in parallel. These included testing simvastatin-eluting AMCA membrane (“SMV” group, 5 animals), hMSC-coated simvastatin-eluting AMCA membrane (“SMV-MSC”, 5 animals), and AMCA membrane alone (“AMCA”, 7 animals control group). In these experiments, the animals had only one random forelimb operated.

**Scheme 1:**
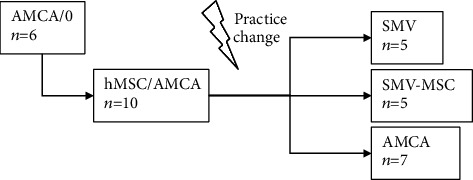
The experiment design.

In total, 33 animals were treated over these five experiment arms, with 49 limbs tested. The study scheme is presented below in Scheme 1.

### 2.2. Polymeric Membrane Preparation

Membranes were prepared using a solvent casting technique [41]. Briefly, a solution containing the polymer (AMCA, Eudragit® RL, obtained from Röhm Pharma, today Evonik Inc.), plasticizer (PEG 400, various sources), optional simvastatin, and ethanol (Frutarom, Haifa, Israel) was cast into Teflon® molds (round flat plates, inner diameter 9.6 cm), and the solvent was allowed to evaporate overnight at 37°C. Simvastatin was received as a gift from Taro Pharmaceutical Industries, Israel.

The membranes that were used were prepared and characterized as previously described [34]; the neat membranes contained 85%wt of AMCA, and 15%wt of plasticizer. The membranes were cast from 10 mL of 4.45%wt solution in ethanol, which resulted in membranes being 180 ± 10 *μ*m thick. Simvastatin-eluting membrane contained 20%wt of simvastatin and a similar composition of plasticized polymer (70%wt of AMCA and 10%wt of PEG400 as plasticizer). Both membranes had comparable mechanical properties after priming in phosphate-buffered saline (see below). The adhesion of hMSC to simvastatin-eluting membrane and their proliferation were similar to the previously reported neat membrane (ibid). Simvastatin was gradually released over 72 hours under sink conditions, i.e., at the artificial testing conditions when the solubility of the drug in the tested medium does not limit the amounts released from a dosage form. Given a poor solubility of simvastatin (about 6 ppm at pH 6.8 (Krishnam [42])) and low lymph turnover at the vicinity of the defect, the release duration of simvastatin in vivo was expected to cover for a significant part of the trial period.

### 2.3. Sterilization and MSC Coating of the Membranes

Membranes were washed in phosphate-buffered saline (PBS, Biological Industries, Beit HaEmek, Israel) for 24 hours and then sterilized with UV irradiation for 2 hours in biological hood. The membranes were then cut to a size according to the surgical requirements (ca. 1.5 cm × 1.5 cm), to be trimmed, if needed, during the implantation.

Human mesenchymal stem cells were harvested from discarded tissue from patients undergoing total hip replacement surgery [43] under informed consent and approval of Helsinki Ethics Committee of Hadassah Medical Center. The cells were maintained in low-glucose Dulbecco's modified Eagle medium (DMEM), supplemented with 10% of heat-inactivated fetal bovine serum, penicillin/streptomycin (100 U/*μ*g per mL, respectively), and glutamine (all by Biological Industries, Beit HaEmek, Israel). To coat the membranes, cells were incubated on the surface of washed membrane piece, 1 × 10^6^ cells in 1 mL of medium spread on the surface of the membrane, for 6 hours at 37°C and humid 5% CO_2_ atmosphere, followed by careful 20-fold dilution with medium to final culturing conditions for additional 24 hours.

### 2.4. Surgical Procedure

The rabbits were anesthetized by a veterinarian using 4 mg/kg IM injection of diazepam. Anesthesia was maintained with subcutaneous ketamine/xylasin (1.5 mL ketamine 10% + 0.5 mL xylasin 2%, per 3 kg of weight). Postoperative subcutaneous carprofen (1.5 mg/kg) and lignocaine were administered for analgesia, repeated every 12 hours for 3 days. The forelimbs were shaved and sterilized. An anterior Henry approach was used to expose the middle third of the radius. Following dissection and periosteal elevation, proximal and distal osteotomies were performed and ca. 1 cm segmental radial defect was created. The periosteal membrane sleeve was resected as well. After careful hemostasis, the defect was irrigated and washed with PBS to remove the hematoma from the defect site. This was done to ensure the least possible spontaneous recovery, considering merely eight-week follow-up. The preconditioned AMCA membrane was wrapped around the defect, by folding it into a cylinder, inserting over the osteotomy gap edges of the resected radius, and secured by Dermabond (Ethicon, Johnson and Johnson). The fascia was sutured with a Vicryl 3-0 suture, and the skin was sutured with Ethilon 4-0 continuous sutures. No fixation was deemed necessary due to intact weight-bearing ulna in this animal model. Subsequent to the surgical procedure, the tested animals were monitored on a daily basis for body weight, signs of dehydration, and pain control. The rabbits were sacrificed 8 weeks after surgery.

### 2.5. Radiological Evaluation

Anteroposterior and lateral radiographs of the tested limbs were obtained at time 0, 2, 4, 6, and 8 weeks (41 kV, 1.2 mas). The data was calibrated using the diameter of the olecranon process at its narrowest point as a reference, defined as 10 mm. The measurements were carried out using an OsiriX DICOM viewer (OsiriX Imaging Software).

### 2.6. Microtomographic Evaluation

After 8 weeks, the osteotomy regions were disarticulated and excised. The disarticulated bone specimens were scanned in a micro-CT 40 apparatus (Scanco Medical AG, Switzerland) at 36 *μ*m voxel size. Segmentation was performed semiautomatically, using Amira software (v. 5.3.3), with automatic skeletonization. The bone volume, total volume, bone surface area, trabecular number, and mean trabecular radii were measured. Based on the segmentation, a three-dimensional visualization was calculated using a Surfgen™ module.

### 2.7. Histology Assessment

The specimens were then dehydrated in alcohol solutions of increasing concentration, cleared in xylene, and embedded in polymethylmethacrylate (PMMA) resin. Bone decalcification was not conducted. The specimens were then sliced into 30 *μ*m thick slides and stained with modified paragon stain for analysis. Using a microcutting and grinding technique adapted from Donath and Breuner [44], a single longitudinal histological section per defect site was obtained. A pathologist experienced with bone pathology evaluated the bone samples.

### 2.8. Statistical Analysis

Statistical calculations were performed at a significance level of *α* = 0.05. Power was calculated when feasible. Data sets were evaluated with Dixon's *Q* test and Grubb's *Z*-score for outliers. The radiographic defect recovery was evaluated by evaluating the filled callus area of the defect at each time point. The crossover experiments were evaluated with paired Student's *t*-test and justified with McCulloch-modified Pitman's test for homoscedasticity. As hMSC/AMCA group data showed leptokurtic tendency, it was log-transformed, which furnished a slightly leptokurtic homoscedastic set. Parallel groups were evaluated with ANOVA modified with Welch's amendment for heteroscedasticity, with post hoc Tukey's test. Calculations were performed with Microsoft Excel software.

The data of the follow-up experiments were then pooled, converted to a percentile of the defect recovery for better normalization, and fitted to a mixed-effect model [45] using JMP® Pro 13.2 software. To accommodate the different approaches, i.e., to justify the pooling of the data from animals operated on a single limb with the data obtained from animals operated under older ethical practice, the model used the treated animal numbers as random effect, nesting within the number of operated limbs. Time, simvastatin presence, and hMSC presence were chosen as independent factors.

## 3. Results

### 3.1. The Effect of AMCA Membranes in Bone Defect Filling

In order to test the effectiveness of AMCA-based barrier membrane in accelerating bone defect recovery, a critical-size radial defect was generated in rabbits with both forearms operated. Whereas one defect was treated by secluding it with the AMCA membrane, the contralateral defect served as the control. The defects were evaluated periodically by radiography for the regenerated callus area. This was found indicative of bone regeneration, as demonstrated by the micro-CT and histology after 8 weeks.

**Figure 1:**
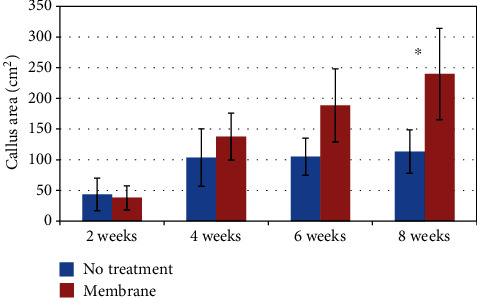
AMCA membrane facilitates significantly higher callus formation as compared to no treatment. The graph shows radiographic callus area over time (mean ± standard error of mean (SE, *n* = 6)). ^∗^*P* < 0.05.

The radiology data for the time points of 2, 4, 6, and 8 weeks of the callus area in the defect zone of the treated and untreated defects are shown in Figure 1. A gradual increase in the callus area with time is seen that is more pronounced in the AMCA-treated group, with statistically significant difference between the groups at week 8 (*P*(*t*) = 0.0498, 1 − *β* ≈ 49%). It is noteworthy that upon removal of an outlier identified with a *Z*-score of over 55*σ*, more powerful statistical results (*P*(*t*) = 0.0167, power ≈ 94%) were furnished profoundly, at a small sample of five animals and despite large apparent variability. In the figure, discordant data point is included.

After 8 weeks, disarticulated bones (radius and ulna) were scanned with micro-CT. The bone volume and callus surface area normalized to total volume were significantly higher in the membrane-treated limbs vs. no-treatment limbs (0.72 vs. 0.21 (*P* < 0.05) and 5.25 mm^−1^ vs. 1.01 mm^−1^, respectively (*P* < 0.05)). The number of bone trabeculae was also significantly larger in the membrane-treated limbs (14,673 vs. 3,033 trabeculae in the control limbs (*P* < 0.05)). However, mean trabecular radii did not vary between the two groups (0.97 vs. 0.85 *μ*m).

**Figure 2:**
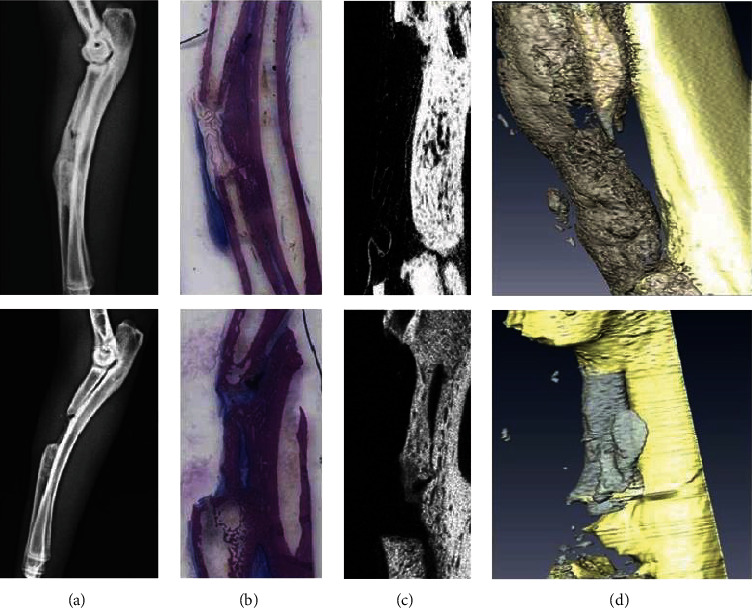
Callus formation after 8 weeks of treatment with AMCA membrane. Representative images of the X-ray, histology, *μ*CT slice, and 3-D visualization (a–d) are shown, treated (top panels) and untreated bones (bottom panels).

Additionally, histologic staining analyses of midsagittal ulnar-radial sections were performed to visualize the membrane. In the control group, specimens showed variable outcomes with one of the sites characterized by a marked cancellous bone growth in radioulnar space, mineralized cartilage, and radioulnar bone fusion: a radioulnar bone reaction which resulted in ulnar periosteal bone growth with signs of a bone bridge between the ulna and the radius. The cortices were not formed. In the AMCA group, a variable bone-healing outcome was observed. A marked cancellous bone growth with slight signs of scattered haversian formation was observed, but no complete bone fusion was observed. However, the level of bone growth towards fusion was greater as compared with the control group. The cortices were not formed. Although the membrane was not clearly identified (the polymer is soluble in alcohol), its limits were easily delineated. No evidence of cell-mediated material degradation was observed. A thin rim of quiescent macrophages was detected along the membrane surface in some areas. In general, the AMCA group of osteotomies had more massive bone development than the no-treatment group. Juxtaposition of similar-thickness slices of histology and *μ*CT and radiographs (Figure 2) of both the AMCA group and no-treatment group reveals consistent morphology and mineralization pattern. After 3-dimensional reconstruction of the CT slices, similar new bone growth as seen on the radiographs is observed.

### 3.2. The Effect of Simvastatin and hMSC Together with the AMCA Membranes

To investigate further the influence of factors that could possibly improve the outcome of the bone regeneration, the membranes were modified, by incorporating into them simvastatin that was released in a controlled manner and by seeding on the membrane hMSC, or by both modalities. Neat AMCA reference group was used as control. The defect healing was followed, as before, by radiography every 2 weeks up to 8 weeks.

Surprisingly, no significant difference was observed in the radiographic callus area in the defect, between animals treated with membranes coated with cells and those treated with the neat membranes, at either time point. Moreover, a statistically nonsignificant trend was observed with the cell-coated membrane to the poorer radiographic callus area in the defect than with the neat membrane.

In the parallel groups, the callus area was significantly higher, in the group treated with simvastatin-eluting hMSC-coated membrane and the neat AMCA membrane, but only at the time point of 6 weeks. The defect recovery values in the groups converged towards week 8 with no significant difference between all groups at that time point.

The data was then pooled and fitted to the mixed-effect model, as described in Materials and Methods. The obtained model parameters are summarized in Table 1. The model showed a high correlation coefficient (*r*^2^ = 0.80, *P* < 0.0001) and statistical significance for the factors of simvastatin elution and for time, as standalone factors (i.e., affecting the system by presence), which means that in the presence of simvastatin, the bone regeneration is accelerated, and it also proceeds spontaneously, with time. The model also showed significance of the interaction of hMSC (“cells”) and time (i.e., the effect of cells present over time), meaning that the effect of the cells in the beginning of the study and in the end is different, and of triple interaction (i.e., the effect of the drug and cells present over time). The model also showed a statistically significant random effect component, indicating that some of the bone-regeneration results might arise from the animals themselves and from the unaccounted noise. However, there were five animals that showed the statistical significance beyond the random effect: two of hMSC/AMCA, one of SMV-MSC, and two of AMCA. Removing the discordant data relieved the concern but did not change the overall picture, albeit slightly decreased the *r*^2^ value (0.73).

**Figure 3:**
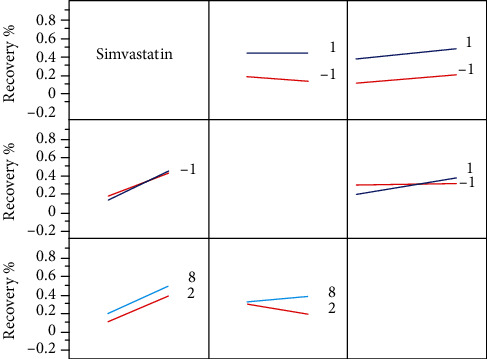
Interaction profiles of the tested parameters. The recovery % indicates the bone regeneration value; the numerals 1 and -1 indicate the presence and the absence of the charted factor, respectively; and the digits 2 and 8 indicate the time as measured in weeks.

An interaction profiler plot of the factors is given in Figure 3. For categorical variables, the numerals 1 and -1 indicate the presence or the absence of the factor, and for time (continuous), 2 and 8 refer to these time points.

It can be readily observed from Table 1 and Figure 3 that the presence of simvastatin is the strongest factor of the model after the intercept which indicates the tendency of the model to heal itself to a certain degree. Time, which was taken as a continuous parameter, is the second most significant factor, although by far less influential. Intriguingly, the interaction between time and simvastatin was absolutely nonsignificant, as evidenced by almost parallel lines, which means the effect of simvastatin was similar at week 2 and at week 8. Conversely, an interaction between hMSC (“cells”) and time is a significant one, showing that the effect of hMSC in the beginning is negative (the declining line) and at the end of the experiment positive (raising line). It can also be seen from Figure 3, that although no significant interaction was observed between the drug and hMSC, the positive effect of the stem cells was more pronounced when simvastatin controlled-release membrane was present (steeper slope). Finally, the triple interaction between the prolonged presence of cells and simvastatin has shown an additional incremental effect with modest statistical significance.

## 4. Discussion

We have previously demonstrated [34] that hMSCs adhere, proliferate, and can differentiate into osteoblasts, when cultured on a polycationic pharmaceutically acceptable AMCA membrane. In this communication, we demonstrate that these membranes may become a valuable tool in assisting bone regeneration *in vivo*. Importantly, critical radial defects in rabbits heal significantly better in the presence of an AMCA membrane than without treatment. The results were achieved despite relatively high noise and a very modest number of animals (*n* = 6), with very convincing statistical power of over 0.94. The membrane facilitated bone growth, indicated by significantly higher radiological area of new bone formation, and bone volume, bone surface area, and new bone trabecular number, as shown by micro-CT scans, and prevented radioulnar synostoses.

It is true that the critical defect created in the present study was smaller than some published recommendation [40] of 1.4 centimeters for this model; however, the complete removal of fracture hematoma contributes to the criticality of the bone defect at smaller sizes, particularly in the short follow-up period of only 8 weeks. The tendency of the rabbit bone defects to heal is a well-known concern (which was also reflected in our parametric analysis results); therefore, removing fracture hematoma allowed demonstrating the effects that could have otherwise been masked by spontaneous healing at shorter defect size.

Conversely, no effect was observed for the presence of hMSC on the membrane in the crossover study with a neat AMCA membrane. A trend for negative influence of the stem cell presence on the bone defect regeneration was observed. The finding is consistent with the anti-inflammatory properties of hMSC [8], which may then mediate the reduction of the natural fracture healing processes. This explanation is partly supported by the reduced edema in the MSC-coated membranes (data not shown). We assume that the cells, once adhered, remained on the membrane; their further fate after the implantation is in the realm of speculation. With numerous studies demonstrating the positive effect of the MSC on the fracture healing (much of it were used in scaffolds though), it may only be assumed that with a barrier membrane in place to seclude the fracture area, the biology may be different with no other tissues involved.

The use of hMSCs in the present study is justified by the animal species used for the present study. Laboratory New Zealand rabbits do not come as a genetically homogeneous population, and there may be concerns in using xenografts with significant HLA mismatch. Human MSCs, however, have been described as immunoprivileged, possess immunosuppressive properties, do not stimulate preactivated lymphocytes, suppress their proliferation, and integrate successfully into host tissue after xenografting in an immunocompetent animal model [46]. Although xenograft MSC transplants may produce inferior results in comparison with autografts under certain conditions [46], the availability of xenografts and the animal welfare justifies their use over autografts.

Simvastatin, on the other hand, has shown positive influence on the bone regeneration, but only on the pooled data with high number of animals from several studies. The data showed that the controlled release of simvastatin is by far the most important factor affecting positively the defect recovery outcome. This is quite in line with the BMP-like properties of the drug, and bone morphogenic proteins have been very frequently used to promote bone regeneration. The advantage of a small molecule over a protein, however, is quite appreciable, and it includes stability, better penetration ability by passive diffusion, and the ready availability of this quite old drug by an established chemistry. No significance for the interaction between time and simvastatin could be attributed to controlled-release formulation or to the fact that the involvement of BMP2 in the fracture healing process is limited to the acute inflammation stage [47], which occurs early in the time course of the process.

The biological underlying of mathematical interaction observed between the presence of the hMSC and the time parameter is an interesting question. It may be assumed that at the early stages, the uncommitted stem cells may indeed decrease the reparatory processes mediated by the inflammation, but once committed (the process that takes time and does occur in cultured hMSC on simvastatin-eluting membranes; data not shown), they improve the healing outcome. This hypothesis is further supported by the effect seen with simvastatin, wherein the conversion is probably occurring earlier and therefore, the slope of the effect graph in the interaction plot (Figure 3) is steeper. A negative sign seen for (nonsignificant) influence of the hMSC per se may serve a mild warning against indiscrete use of hMSC in bone regeneration, particularly when no scaffolds or fracture hematoma are used, to assist osteoconduction.

On the other hand, comparing head-to-head with the staple statistical methodology, the effect of added simvastatin, either alone or on MSC-coated membrane, results in inconclusive data, due to high variability and relatively low number of subjects in each group for such comparison. The difference seen at week 6 between the simvastatin-eluting AMCA membrane coated with hMSC and neat AMCA membrane did not last to week 8, due to the convergence of the effects. The AMCA membrane by itself was shown a significant factor ensuring enhanced bone regeneration, and a further effect of either factor could not be unequivocally demonstrated. The data obtained in the follow-up experiments was rather noisy, due to the change of ethical practice between the experiments and high intersubject variability, which contributed, among other factors, to mostly inconclusive results obtained with standard statistical analysis.

Statistical significance can sometimes be more readily obtained for an observed difference using a higher sample size, resulting in more degrees of freedom. Therefore, pooling all the results and looking for significant effects in multivariate models were more successful approaches. Conversion of the data to a percentile of defect recovery, rather than the radiographic defect filling, allowed better normalization of the values through various transformations. As the pooled data that was eventually used for the parametric analysis was derived from a population with inherent variability of the operated limbs, the statistical model was used to account for the variability. The animals' identities were used as random effect, and the number of operated limbs was used as a nested factor, which is probably the most appropriate treatment of the presented data.

Radioulnar synostoses that were observed in some specimens of the control group demonstrate again that the intervention of the surrounding tissues is not always beneficial to proper bone regeneration. The ability of the AMCA membrane to prevent the involvement of the neighboring bone indicates that these membranes, at least to some extent, are able to confine the defect site and allow the natural processes of bone regeneration to better exert their full capacity. In addition, the use of AMCA membrane facilitated bone regeneration, emphasizing the advantage of this strategy. Therefore, the potential of the barrier membranes and AMCA membranes in particular in the treatment of fractures with high-risk of nonunion should be further explored. The versatility of these membranes and the possibility to contain and controllably release small bone-active molecules, like simvastatin, can be advantageously exploited to further improve the clinical outcomes of bone regeneration.

## 5. Conclusions

In this communication, we have demonstrated that a barrier membrane based on known pharmaceutical polymer, AMCA, improved bone regeneration in rabbit critical radial defect. The addition of simvastatin to the treatments significantly increased bone defect recovery, but the effect of hMSC is less pronounced. The effect of hMSCs committed to osteogenesis by controlled release of simvastatin may have an additional positive influence on the defect healing.

Our results suggest that the potential of AMCA membranes should be investigated, particularly for complicated fractures, where the fracture hematoma is naturally formed and never removed. Our results convincingly demonstrate that controlled-release simvastatin dosage forms for local delivery should be further investigated for acceleration of bone fracture healing.

## Figures and Tables

**Table 1:** Mixed-model parameter estimates; star (∗) indicates statistical significance.

Term	Estimate	Std error	DF Den	*t* ratio	Prob > ∣*t*∣
Intercept	0.2012025	0.035087	60.83	5.73	<0.0001^∗^
Simvastatin	0.137325	0.026433	22.23	5.20	<0.0001^∗^
Cells	-0.0065771	0.021647	26.76	-0.30	0.7636
Simvastatin∗cells	0.0116413	0.021647	26.76	0.54	0.5952
Time	0.0170366	0.004625	95.93	3.68	0.0004^∗^
Simvastatin∗time	0.001747	0.004625	95.93	0.38	0.7064
Cells∗time	0.013256	0.004618	95.86	2.87	0.0050^∗^
Simvastatin∗time∗cells	0.0099246	0.004618	95.86	2.15	0.0342^∗^

## Data Availability

The data used to support the findings of this study are available from the corresponding author upon request.
